# PS-26 - EVOLVING MPOX EPIDEMIOLOGY IN ENGLAND, 2023–2026

**DOI:** 10.1093/pubmed/fdag046.068

**Published:** 2026-07-09

**Authors:** Celine El Hakim, Miss Holly Fountain, Mrs Katy Sinka

**Affiliations:** Blood Safety, Hepatitis, STIs & HIV Division, UK Health Security Agency (UKHSA); Blood Safety, Hepatitis, STIs & HIV Division, UK Health Security Agency (UKHSA); Blood Safety, Hepatitis, STIs & HIV Division, UK Health Security Agency (UKHSA)

## Abstract

**Background:**

Following the 2022 mpox outbreak, case numbers in England remained low. However, an increase in November 2025 preceded a sharp increase in early 2026. Several factors contributed, evidence of an evolving epidemiological picture.

**Objective:**

Describe epidemiological changes in mpox cases reported in England, focusing on transmission dynamics, demographics, and viral clades.

**Methods:**

Diagnoses (01/01/2023-28/02/2026) were extracted from the UKHSA mpox surveillance line list (Second Generation Surveillance System), enhanced with Health Protection Teams questionnaire data and HPZone/CIMS records. Descriptive analyses by viral clade examined region, travel history, vaccination status and demographics.

**Results:**

Following low and stable clade IIb reporting at an average of 21 cases/month in 2024-2025, an increase was observed in 2026, With 56 cases reported in January rising to 110 in February. Where data was known, a greater proportion of 2026 cases were UK-acquired versus imported (81% vs 58% in 2024–2025), indicating increased local transmission. Proportion vaccinated increased slightly/non-significantly from 48% (2024–2025) to 53% (2026), although proportion of UK-acquired cases vaccinated in 2026 stayed equal (47% vs 48% respectively). Cases outside London increased in 2026, notably in the North West, with 48% of cases reported outside London versus 29% in 2025 and 21% in 2024.

Clade Ib cases increased from an average of 1/month in 2025 to 15 in 2026 (13 in February). An epidemiological shift was observed, mirroring patterns in Europe, from predominantly travel-associated cases in heterosexual individuals linked to endemic countries to almost exclusively non-travel-associated cases among gay, bisexual, and other men who have sex with men.

**Conclusion:**

The increase in cases in England is accompanied by evolving epidemiology. The expansion of clade IIb beyond London and shifts in clade 1b demographics highlight the need for continued surveillance to detect changes early, monitor vaccine effectiveness and inform overall understanding of mpox.

